# Evidence on interventions that target prescribing appropriateness in older adults attending the emergency department and other acute care settings: a scoping review

**DOI:** 10.1007/s11096-026-02170-8

**Published:** 2026-06-18

**Authors:** Lauren Fernandes, Daniel McCarthy, Claire McCormack, Aoife Leahy, Anne Harnett, Abdirahman Mohamed, Ahmed Gabr, Margaret O’Connor, Rose Galvin

**Affiliations:** 1 School of Allied Health, Faculty of Education and Health Sciences, University of Limerick, Limerick, Ireland; 2 School of Medicine, Faculty of Education and Health Sciences, University of Limerick, Limerick, Ireland; 3 University Hospital Limerick, Limerick, Ireland; 4 Ageing Research Centre, Health Research Institute, University of Limerick, Limerick, Ireland

**Keywords:** Acute geriatric unit, Aged, Emergency service, Hospital, Medication review, Potentially inappropriate medication list, Pharmacists

## Abstract

**Introduction:**

Potentially inappropriate prescribing (PIP) has been associated with various adverse clinical outcomes, particularly in the context of ageing, multimorbidity and polypharmacy. Despite growing interest in front door frailty initiatives in the emergency department (ED) and acute geriatric units (AGUs), no review has focused specifically on interventions targeting PIP, as defined by validated criteria, across both ED and AGU settings.

**Aim:**

This scoping review aimed to map the evidence on interventions addressing PIP in older adults attending EDs or AGUs, identify evidence gaps and highlight areas for future research.

**Method:**

This scoping review was conducted in accordance with Joanna Briggs Institute (JBI) methodology and reported using the Preferred Reporting Items for Systematic Reviews and Meta-Analyses extension for Scoping Reviews (PRISMA-ScR) Checklist. Medline, Embase, CINAHL Ultimate, Web of Science, Cochrane CENTRAL and grey literature sources were searched from 1991 to 2025 for studies evaluating interventions targeting PIP, defined using validated criteria, in adults aged ≥ 65 years attending the ED or AGUs.

**Results:**

Our search returned 8643 results. Twenty-one studies were identified for inclusion, four of which were randomised controlled trials. The 18 interventions identified encompassed pharmacist-led medication reviews, clinical decision support systems (CDSS), educational/academic detailing programmes or combined approaches. While PIP was frequently a primary outcome measure, few studies reported clinical outcomes or explored prescriber adherence and experiences of older adults and prescribers.

**Conclusion:**

Pharmacist-led medication reviews, CDSS and educational/academic detailing were the main intervention approaches identified. Evidence was heterogeneous and focused mainly on prescribing-related outcomes, with limited assessment of clinical outcomes, prescriber adherence, communication pathways and stakeholder feedback. Future studies should incorporate longer-term follow-up and evaluate patient-centred and implementation outcomes.

**Supplementary Information:**

The online version contains supplementary material available at https://doi.org/10.1007/s11096-026-02170-8.

## Impact statements

- This scoping review maps the evidence on interventions targeting potentially inappropriate prescribing in older adults attending emergency departments and acute geriatric units.
- Existing interventions most commonly involved pharmacist-led medication review, clinical decision support systems and educational/academic detailing.
- The findings support greater pharmacist involvement in emergency department and acute geriatric unit pathways, alongside clearer communication with prescribers, general practitioners, patients and caregivers.
- Further studies should include clinical outcomes, longer-term follow-up, prescriber adherence and stakeholder feedback.

## Introduction

The proportion of the world’s population comprising older adults (aged ≥ 65 years) is expected to double between 2019 and 2050 to an estimated 1.5 billion [1]. This will be accompanied by an increase in multimorbidity (two or more chronic conditions), which affects more than half of older adults [2]. Polypharmacy (the concurrent use of five or more medications) is also common, affecting 26.3–39.9% of older adults in European populations [3]. Older adults account for 15.9–26.9% of emergency department (ED) attendances [4–6], with visit rates increasing with age [4].

Polypharmacy is the main determinant of potentially inappropriate prescribing (PIP) [7], which encompasses the overuse, underuse and misuse of medications. PIP has been defined using validated criteria such as the Screening Tool of Older Persons' Prescriptions/Screening Tool to Alert doctors to Right Treatment (STOPP/START) criteria [8], the American Geriatrics Society (AGS) Beers Criteria [9] and the Medication Appropriateness Index [10]. PIP is associated with adverse outcomes including hospital admission, adverse drug reactions, functional decline and falls [11, 12]. Rates of PIP vary internationally and appear inversely correlated with national economic level [13], potentially reflecting differences in healthcare access and the development of prescribing criteria.

While several systematic reviews have examined interventions to reduce PIP in hospital, long-term care and community settings [14–16], no review has focused specifically on interventions targeting PIP, as defined by validated criteria, across both ED and AGU settings. This is despite growing interest in front door frailty initiatives [17–20] and ED-based comprehensive geriatric assessments (CGAs) to minimise morbidity, mortality and nursing home admission in older adults [19, 20]. Given the link between PIP and frailty [21], this represents a potential missed opportunity for intervention.

## Aim

This scoping review aimed to map the evidence on interventions addressing PIP in older adults attending EDs or AGUs, identify evidence gaps and highlight areas for future research. It described intervention characteristics, summarised reported outcomes, and explored prescriber adherence and stakeholder feedback.

## Method

This review followed Joanna Briggs Institute (JBI) methodology and Preferred Reporting Items for Systematic Reviews and Meta-Analyses extension for Scoping Reviews (PRISMA-ScR) reporting guidance [22]. The protocol was prospectively registered with the Open Science Framework on 14 July 2023 (https://osf.io/49kd8) [23].

### Search strategy

Electronic databases (Medline, Embase, CINAHL Ultimate, Web of Science and Cochrane CENTRAL) were searched for studies published from 1991 (the year of publication of the first known Potentially Inappropriate Medication [PIM] list) to 17 August 2025 in English and involving human participants, with inclusion restricted to English-language studies due to feasibility and resource constraints. Grey literature sources (BASE, Google Scholar, ProQuest, ClinicalTrials.gov and the WHO Trial Register) were also searched.

Search terms were developed using the population-concept-context (PCC) framework and tailored to each database. The Embase search strategy is provided in Appendix 1 in the Supplementary Material.

### Eligibility criteria

Studies were included if they (1) involved adults aged ≥ 65, or mixed-age populations where data for adults aged ≥ 65 could be extracted separately; (2) evaluated an intervention targeting PIP, defined using validated criteria, such as STOPP/START or Beers; (3) were conducted or initiated in the ED or AGU setting and (4) employed randomised or non-randomised primary research study designs.

Studies conducted exclusively in community, long-term care, general inpatient or non-acute care settings were excluded.

### Rationale for context

The ED represents a key point for initiating interventions targeting PIP, particularly in the context of acute presentations reflected in tools such as STOPP/START. While it may not always be feasible to take an intervention to completion in the ED, this setting provides an opportunity for initiation and continuation across care transitions.

For this review, AGUs were defined as dedicated acute hospital units or services providing specialist geriatric assessment and management for older adults during acute illness or hospital admission, including acute geriatric, acute care for the elderly and geriatric emergency medicine units where care was underpinned by interdisciplinary CGA or geriatrician-led multidisciplinary management [24]. AGUs were included because they differ from general geriatric inpatient wards, and prior reviews studying interventions addressing PIP in geriatric inpatients typically have not included this population [25]. Pharmacists are not universally considered core members of the CGA team [26], and medicines optimisation cannot be assumed to occur routinely in this setting.

### Selection of sources of evidence

Screening was conducted independently by three reviewers (LF, DM, CM) at title/abstract and full-text stages. Discrepancies were resolved through discussion and input from a fourth reviewer (RG).

### Data charting process & analysis

Extracted data included study characteristics, population, setting, intervention type, PIP criteria and outcomes (Appendix 2). Data charting was performed by LF using Covidence [27] and checked by AL.

Results were reported using a PRISMA flow diagram [28], tables, figures and narrative summaries. Interventions were classified according to the Cochrane Effective Practice and Organisation of Care (EPOC) taxonomy [29], and inductively categorised into pharmacist-led, educational/academic detailing and clinical decision support systems (CDSS). Detailed study-level results are presented in Appendix 2 for descriptive reporting rather than effectiveness comparisons.

## Results

### Study selection

**Fig. 1:**
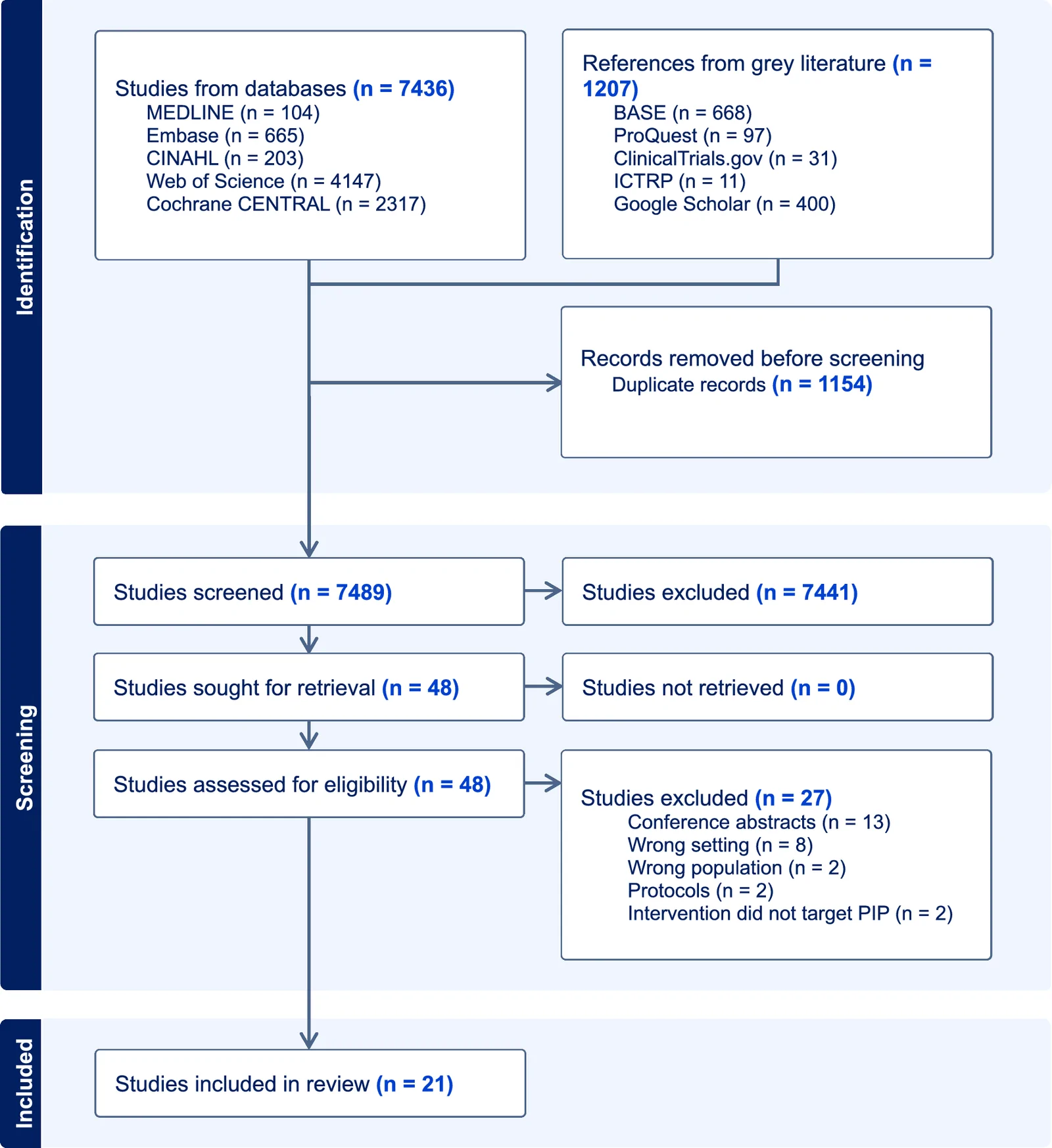
Preferred reporting items for systematic reviews and meta-analyses (PRISMA) flow diagram of study selection process

Electronic searches identified 8643 records. After removing 1154 duplicates, 7489 records were screened by title and abstract. Of 48 full texts assessed, 21 studies were included (Fig. 1).

### Characteristics of included studies

Ten studies originated from the United States [30–39], eight from European countries [40–47] and the remainder from Australia and Taiwan [48–50]. Four randomised controlled trials (RCTs) were identified [34, 44–46], with other study designs described in Appendix 2.

### Intervention characteristics

Eighteen unique interventions were described across 21 studies (Table 1). Using the EPOC taxonomy [29], most were classified as delivery arrangements (n = 13), with one implementation strategy and the remainder comprising a combination of both.

**Table 1:** Intervention characteristics

Study ID	Intervention summary and delivery	Intervention elements	PIP tool/ criteria	EPOC classification
Stevens 2015 USA [30]	**Setting:** VA EDs **Deliverer(s):** Interdisciplinary implementation team including ED physicians, geriatricians and clinical pharmacists **Intervention:** Three-component EQUIPPED quality improvement intervention comprising: provider education through didactic teaching and academic detailing; EHR-based CDSS with geriatric pharmacy order sets, dose adjustments and prescribing education; and audit/feedback via prescriber reports, peer benchmarking, academic detailing and/or informatics dashboards	Educational / Academic detailing & CDSS	Beers	Implementation strategies & Delivery arrangements
Stevens 2017 USA [31]				
Vaughan 2021 USA [32]				
Goldberg 2022 USA [35]				
Vaughan 2023, USA [33]				
Vandenberg 2024, USA [38]				
Moss 2019 USA [37]	**Setting:** VA ED **Deliverer(s):** Physician-pharmacist pair **Intervention:** Academic detailing on core geriatric prescribing principles delivered at internal medicine resident rotation start; CDSS implemented later	Educational / Academic detailing CDSS	Beers	Implementation strategies & Delivery arrangements
Terrell 2009 USA [34]	**Setting:** ED **Deliverer(s):** Computerised physician order entry system/CDSS (designed by expert multidisciplinary panel) **Intervention:** Alternatives offered for nine PIMs at ED discharge; physicians could accept or reject recommendations, with reasons documented	CDSS	Beers	Implementation strategies & Delivery arrangements
O’Connor 2021 UK [40]	**Setting:** CDU of an ED **Deliverer(s):** Unclear **Intervention:** Awareness-raising intervention using one email, three presentations and five posters focused on PIMs and PPOs identified at baseline	Educational / Academic detailing	STOPP/ START	Implementation strategies
Spinewine 2007 Belgium [44]	**Setting:** Acute GEM unit **Deliverer(s):** Clinical pharmacist **Intervention:** Medication history, PIP identification and pharmaceutical care plan completed; recommendations discussed with prescribers and communicated to patients/caregivers and GP	Medication review	MAI, Beers, ACOVE	Delivery arrangements
Lozano-Montoya 2015 Spain [41]	**Setting:** AGU **Deliverer(s):** Clinical pharmacist, geriatric MDT **Intervention:** Pharmacist-led drug history and PIP identification on admission; recommendations agreed within MDT, with reasons documented and information provided to patient/caregiver	Medication review	STOPP/ START	Delivery arrangements
Liu 2019 Taiwan [50]	**Setting:** ED **Deliverer(s):** Clinical pharmacist **Intervention:** Computer-assisted medicines reconciliation and PIP/polypharmacy screening; pharmacist reviewed outputs, communicated recommendations electronically to physicians, and followed up rejected recommendations	CDSS Medication review	Taiwanese adaptation of Beers	Delivery arrangements
Santolaya-Perrin 2019 Spain [45]	**Setting:** ED **Deliverer(s):** Pharmacist & emergency specialist **Intervention:** Pharmacist-led medication review to identify PIP. Joint decision reached with an emergency specialist & forwarded to GP by email	Medication review	STOPP/ START	Delivery arrangements
Gutiérrez-Valencia 2019 Spain [42]	**Setting:** AGU **Deliverer(s):** Clinical pharmacist **Intervention:** Medication history, reconciliation and adherence assessment; drug-related problems addressed using oral/written advice, followed by CDSS-assisted medication review and EHR documentation ± verbal communication	CDSS Medication review	STOPP/ START	Delivery arrangements
Houlind 2020 Denmark [43]	**Setting:** ED **Deliverer(s):** Clinical pharmacist, geriatrician **Intervention:** Medicines reconciliation followed by joint medication review with a geriatrician; proposed changes accepted/rejected by geriatrician, then reviewed by treating physician prior to discharge medication list update	Medication review	STOPP/ START MAI and AOU	Delivery arrangements
Farhat 2022 Switzerland [46]	**Setting:** ACE unit **Deliverer(s):** Clinical pharmacist **Intervention:** Electronic STOPP/STARTv2 medication review; recommendations accepted/rejected by treating physician, outpatient physician informed of changes	CDSS Medication review	STOPP/ START	Delivery arrangements
	Setting and deliverer(s) as above. Intervention as above, using the PIM-Check tool		PIM-Check tool	Delivery arrangements
Jovevski 2023 USA [36]	**Setting:** ED **Deliverer(s):** Triage nurse, clinical pharmacist **Intervention:** ISAR-based triage screening triggered EHR alerts to the ED clinical pharmacist for medication review, with recommendations submitted electronically to the patient’s primary care provider	CDSS Medication review	Beers	Delivery arrangements
Atey 2023 Australia [48]	**Setting:** ED **Deliverer(s):** Clinical pharmacist **Intervention:** Early BPMH followed by traditional medication charting	Medicines reconciliation	Beers	Delivery arrangements
	**Setting:** ED **Deliverer(s):** Clinical pharmacist, medical officer **Intervention:** Early BPMH followed by clinical discussion with medical officer leading to a joint treatment and medication charting plan i.e. PPMC	Medication review	Beers	Delivery arrangements
Reilly 2024 Australia [49]	**Setting:** ED **Deliverer(s):** Clinical pharmacist **Intervention:** Medication review including reconciliation, adherence assessment and STOPP/START application; patient education and updated medication list provided, with recommendations sent electronically to GP after ED discharge	Medication review	STOPP/ START	Delivery arrangements
Elder 2025 USA [39]	**Setting:** ED **Deliverer(s):** EHR-based CDS; developed by physician and pharmacist specialists **Intervention:** EHR-based geriatric CDSS for targeted PIMs, using custom order panels for medications ordered during the ED visit and at ED discharge	CDSS	Beers	Implementation strategies & Delivery arrangements
Hellinger 2025 Germany [47]	**Setting:** ED **Deliverer(s):** Clinical pharmacist & treating physician **Intervention:** Pharmacist-led FRID identification and medication review; agreed management plan developed with treating physician	Medication review	Beers & PRISCUS list	Delivery arrangements

*VA* Veterans Affairs, *ED* Emergency department, *EQUIPPED* Enhancing Quality of Prescribing Practices for Older Veterans Discharged from the Emergency Department, *EHR* Electronic health record, *CDSS* Clinical decision support system, *PIM* Potentially inappropriate medication, *CDU* Clinical decision unit, *PPO* Potential prescribing omission, *STOPP/START* Screening Tool of Older Persons' Prescriptions/Screening Tool to Alert to Right Treatment, *GEM* Geriatric emergency medicine, *PIP* Potentially inappropriate prescribing, *GP* General practitioner, *MAI* Medication appropriateness index, *ACOVE* Assessing Care of Vulnerable Elders, *AGU* Acute geriatric unit, *MDT* Multidisciplinary team, *AOU* Assessment of Underutilization, *ACE* Acute Care for the Elderly, *ISAR* Identification of Seniors At Risk, *BPMH* Best possible medication history, *PPMC* Partnered pharmacist medication charting, *FRID* Fall-risk-increasing-drug

Thirteen interventions, across 17 studies, were conducted in EDs [30–40, 43, 45, 47–50], while five, across four studies, were conducted in AGUs [41, 42, 44, 46].

**Fig. 2:**
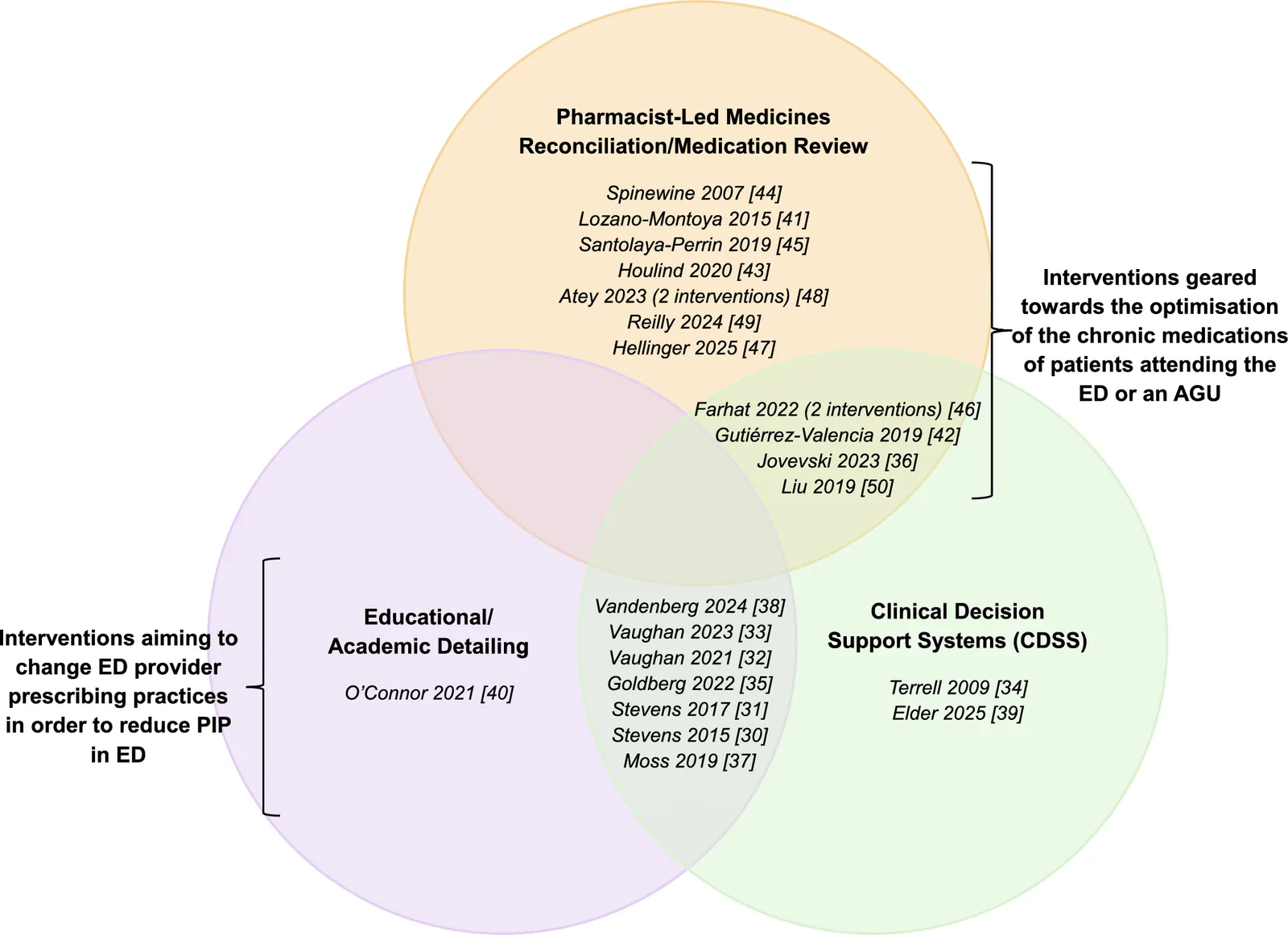
Venn diagram showing studies classified by intervention type. Abbreviations: ED—emergency department; AGU—acute geriatric unit; PIP—potentially inappropriate prescribing; CDSS—clinical decision support systems

Interventions were further classified using two complementary frameworks (Fig. 2). First, interventions were grouped by primary focus: targeting ED provider prescribing practices to reduce PIP, or optimising patients’ chronic medications during an ED or AGU encounter. Second, they were categorised as pharmacist-led medication review, educational/academic detailing or CDSS.

Ten studies evaluated interventions targeting ED provider prescribing practices, comprising educational, CDSS and multifaceted approaches such as the EQUIPPED (Enhancing Quality of Prescribing Practices for Older Veterans Discharged from the ED) programme implemented in Veterans Affairs (VA) Medical Centres in the US [30–33, 35, 37, 38].

The remaining 11 studies evaluated pharmacist-led interventions aimed at optimising chronic medications [36, 41–50], several of which incorporated CDSS components [36, 42, 46, 50].

### Communication of recommendations

**Fig. 3:**
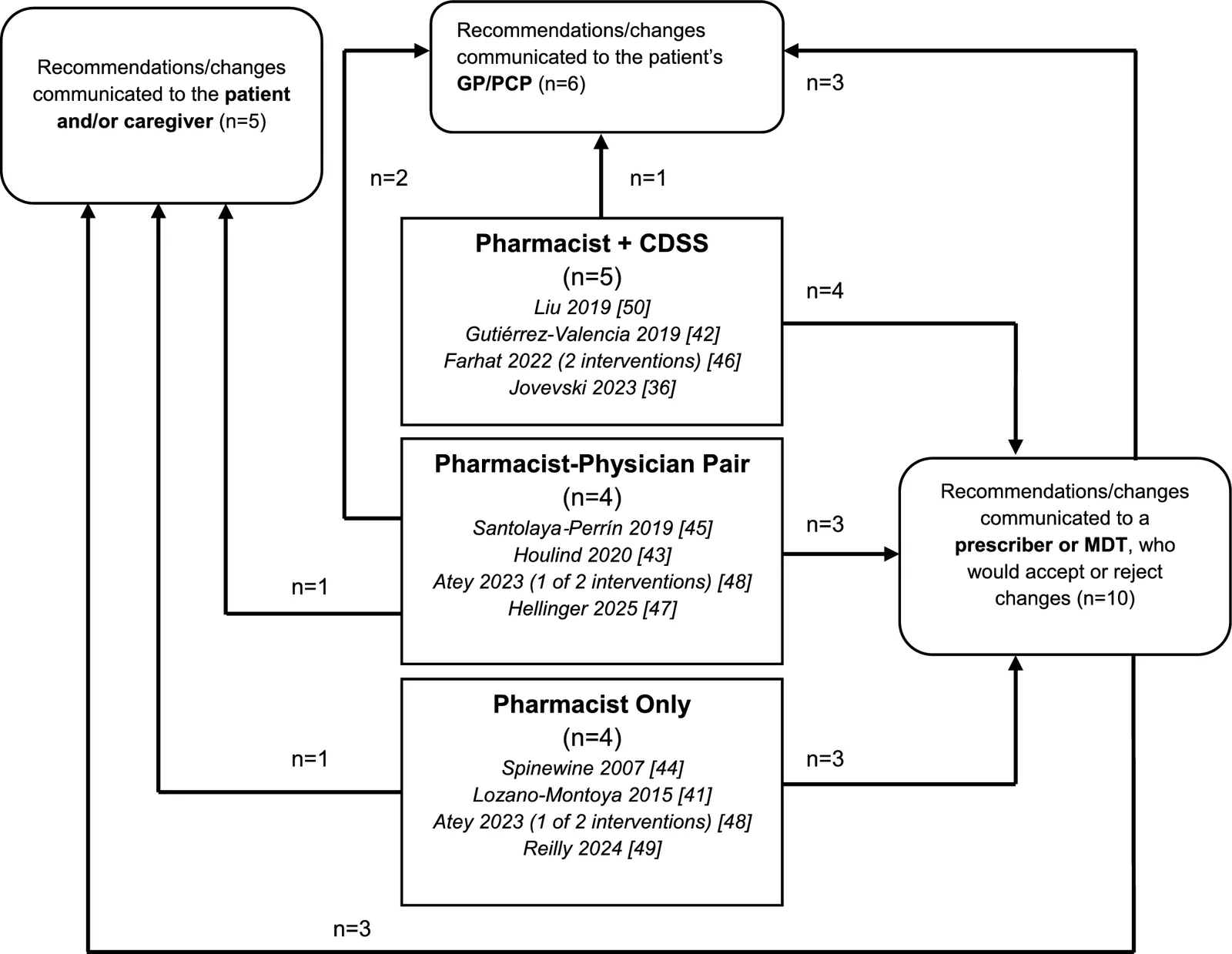
Illustration of flow of communication within prescribing pathways between different stakeholders. Values of *n* refer to the number of interventions, not the number of studies. Abbreviations: GP—general practitioner; PCP—primary care practitioner; CDSS—clinical decision support systems; MDT—multidisciplinary team

Figure 3 illustrates intervention-level communication pathways within the 11 studies evaluating chronic medication optimisation.

Among the 13 interventions in these studies, recommendations were communicated by pharmacists to a prescribing physician in 10 interventions, directly to patients/caregivers in one, and/or to general practitioners (GPs) in two. Overall, six interventions involved communication with GPs, and five provided patients/caregivers with information about medication changes.

### Validated tools to identify PIP

The most common tool to identify PIP was the Beers criteria, used in 14 studies [30–39, 44, 47, 48, 50]. The STOPP/START criteria were used in six of eight European studies [40–43, 45–47]. Other measures included the Medication Appropriateness Index (MAI) [51], Assessing Care of Vulnerable Elders (ACOVE) underuse criteria [52], Assessment of Underutilisation (AOU) index [53], PRISCUS list [47] and the PIM-Check tool [54].

### Reported findings by intervention focus

Findings are summarised descriptively. No formal comparative effectiveness synthesis was undertaken.

#### On PIP via new prescriptions from ED providers

Ten studies evaluated interventions targeting ED prescribing practices [30–35, 37–40]. The only RCT [34], evaluating CDSS, reported a significant reduction in ED discharges associated with new PIP (3.9 to 2.6%; OR = 0.55, 95% CI = 0.34–0.89) as well as a reduction in PIM prescribing (5.4 to 3.4%, *p* = 0.006, OR = 0.59, 95% CI 0.41–0.85). Another CDSS-based study reported significantly higher proportions of targeted PIMs adherent to recommendations both at the point of prescribing and at ED discharge [39]. A quality improvement intervention was also reported to reduce STOPP-defined prescribing errors [40].

Six studies evaluated the multipronged EQUIPPED intervention [30–33, 35, 38]. They reported reductions in PIM prescribing, including a decrease from 9.4% (1.5) to 4.6% (1.0) (RR = 0.48, 95% CI 0.40–0.59, *p* < 0.001) in the earliest study [30]. In two studies, significant reductions in PIM prescribing rates were reported across all four sites [31, 38], with another reporting a reduction in only one of three sites [32]. EQUIPPED was also applied in three non-VA EDs, with an overall reported reduction in monthly PIM rates [35]. A study comparing two EQUIPPED audit/feedback approaches across eight sites reported greater reductions in PIM prescribing with academic detailing than with an informatics-based dashboard [33]. One study reported improved prescribing confidence and reduced PIM prescribing following the educational component [37].

#### On potentially inappropriate chronic medication utilisation

Eleven studies, encompassing 13 interventions, explored pharmacist-led interventions targeting potentially inappropriate chronic medications [36, 41–50]. Overall, studies reported improved prescribing appropriateness.

One RCT reported significant improvements in ACOVE underuse criteria (OR = 6.1, 95% CI = 2.2–17.0) and MAI scores (OR = 9.1, 95% CI = 4.2–21.6), but no difference when using Beers criteria [44].

Partnered pharmacist medication charting (PPMC; Table 1) was reported in another study to reduce the risk of ≥ 1 PIM at ED departure versus the early best possible medication history (BPMH) and usual care groups; although this was not sustained at hospital discharge [48].

In one study, 83% and 69% of older adults had STOPP and START recommendations followed by their GP post-discharge, respectively [49]. Improvements in prescribing appropriateness were reported in another study, with 83% of changes sustained thirty days post-discharge [43]. While 87.0% of STOPP-defined PIMs were discontinued in another study, only 33.5% of START-defined potential prescribing omissions (PPOs) were commenced [41].

One study reported reductions in the number of patients prescribed falls-risk-increasing-drugs (FRIDs) and the median number of FRIDs prescribed at ED discharge [47].

Four studies incorporated CDSS components [36, 42, 46, 50], reporting reductions in rates of PIM prescribing and prescribing omissions, and increased deprescribing rates at follow-up. One RCT reported a significant reduction in PIP in the STOPP/START and PIM-Check arms at 34% and 31% respectively [46].

#### On polypharmacy

Five studies measured changes in medication number following intervention [41–43, 46, 50]. One study reported reductions in polypharmacy and hyperpolypharmacy, although these were not sustained at 30 days post-discharge [43]. In one RCT, the proportion of patients with a decrease in number of prescribed drugs at discharge was reported to be 18.3% for the PIM-Check intervention arm and 12.9% for the STOPP/START arm (*p* = 0.023) [46].

#### On clinical outcomes

Only four studies measured clinical outcomes [36, 44–46], three of which were RCTs [44–46]. Most reported no significant improvement in length of stay, delirium, falls [46] or mortality [36, 44]. One RCT compared two tools to identify PIM, and reported that patients in one arm of the trial had significantly higher activity of daily living (ADL) scores between home and discharge than those in the PIM-Check arm (4.42 vs 3.77; *p* = 0.040). Reported predictors of ADL score improvement included application of STOPP-START criteria and deprescription of central nervous system drugs [46].

Another RCT reported reductions in ED visits and hospital readmissions at three-month follow-up only (rate ratios 0.452, 95% CI 0.222–0.923 and 0.567, 95% CI 0.328–0.983) in the two sites with the highest rates of GP acceptance of prescribing recommendations [45], however, other studies reported no benefit in these outcomes [36, 44, 46]**.**

### Adherence to interventions

Six studies reported prescriber acceptance of recommendations [34, 39, 41, 45, 46, 49], with only two exploring reasons for rejection [34, 41]. Reported GP acceptance rates ranged from 27% to 53% across four sites in one RCT [45], while higher rates (83% and 69% for STOPP and START recommendations, respectively) were reported in a smaller cohort study [49]. Physician acceptance rates were reported to be 32% and 43% in the PIM-Check and STOPP/START arms of an RCT [46], and 43% for CDSS recommendations amongst ED physicians in another study [34].

A non-randomised study identified 284 STOPP and 397 START recommendations following pharmacist-led medication review [41]. After multidisciplinary team consensus, 87.0% of STOPP-defined PIP were discontinued at discharge, compared with initiation of 33.5% of START-defined PPOs. Elder et al. also reported increased adherence to CDSS recommendations at the point of prescribing in ED and at ED discharge [39].

Common reasons for rejection included perceived clinical necessity, medication refill, lack of alternatives, dose reduction in lieu of deprescribing, patient preference, advanced physical disability and potential adverse events [34, 41].

### Stakeholder feedback on interventions

In one study, feedback on a deprescribing intervention was sought from 45 GPs, with low response rates [49]. Three gave positive feedback, while a fourth noted lack of appropriateness in the context of their patient’s past medical history. Another study mentioned anecdotally positive responses by GPs [36].

Pharmacist-led medication review was well-received by 74% (n = 35) of patients in one study [49], with the following reasons provided: first such opportunity; improved symptoms or blood pressure; better understanding of and fewer unnecessary medications. One participant stated, “He explained things and listened to me”. Patient satisfaction reportedly rose from 60.9 to 80% at one-month post-discharge in another RCT [44].

## Discussion

### Statement of key findings

To our knowledge, this review is the first to map interventions specifically targeting PIP, as defined by validated criteria, across both ED and AGU settings. Twenty-one studies were included, including four RCTs [34, 44–46]. Interventions comprised pharmacist-led medication reviews, CDSS and/or educational/academic detailing. Most studies reported reductions in PIP and polypharmacy-related measures, although few assessed clinical outcomes, adherence or stakeholder feedback. Limited prescriber uptake, patient/GP involvement, and clinical outcome data may represent key barriers to improving prescribing appropriateness in acute care settings. All studies were conducted in developed countries, despite higher reported prevalence of PIP in lower-income settings [13].

### Strengths and weaknesses

This review followed JBI guidance, was preceded by a published protocol [23], and included searches of peer-reviewed and grey literature. English-language restriction may have excluded relevant evidence. Quality and risk of bias assessments were not undertaken. Findings were summarised descriptively and were not used to infer comparative effectiveness. All included studies were conducted in developed countries, limiting generalisability.

### Interpretation

PIP is associated with adverse health outcomes [11, 12], yet research targeting this issue in ED and AGU settings remains limited despite increasing emphasis on front door frailty initiatives and ED-based CGA models [17–19]. This suggests a missed opportunity for intervention at this stage of care.

In contrast, a larger evidence base exists outside ED and AGU settings, including systematic reviews of medication optimisation in long-term care [55], medication review in hospitalised older adults [16], deprescribing in community-dwelling populations [15], and PIP interventions in primary care and long-term care populations [14].

A recent review of ED-based geriatric medication safety programmes reported improvements in PIM-related process measures, but limited evidence of clinical benefit [56]. This review extends that literature by mapping ED/AGU interventions, describing communication pathways, prescriber adherence and stakeholder feedback.

The interventions identified in this review may address prescribing appropriateness in complementary ways. Pharmacist-led medication review may help identify PIMs, prescribing omissions and drug-related problems during medicines reconciliation and treatment planning. CDSS may support prescribers at the point of prescribing, particularly for ED discharge prescriptions, while educational and academic detailing interventions may influence prescribing behaviour. These approaches highlight the potential value of pharmacist involvement in medicines optimisation within ED and AGU pathways.

Interpretation of reported improvements was limited by variation in the tools used to assess prescribing appropriateness. STOPP/START identifies both PIMs and PPOs, whereas Beers criteria focus mainly on PIMs. The MAI differs from these explicit criteria as an implicit, judgement-based measure of prescribing appropriateness, while PIM-Check is a prescription-screening checklist for internal medicine patients [8–10, 51, 54]. These differences limit comparability across studies.

Limited communication of prescribing recommendations to patients, caregivers and GPs was an important implementation issue across pharmacist-led medication review studies [36, 41–50]. Pharmacist integration into multidisciplinary teams is a key factor in successful deprescribing interventions [57], and effective communication within the patient-clinician-pharmacist triad influences patients' willingness to consider deprescribing [58].

Evidence regarding clinical outcomes was limited. One RCT reported higher ADL scores in one of its intervention arms [46], while another reported reductions in ED visit and hospital admissions at sites with higher GP acceptance rates, albeit only at the three-month follow-up point [45]. In comparison, medication reviews in the hospital setting likely reduce hospital readmissions and possibly ED reattendance without affecting mortality [16]. Similarly, interventions in long-term care improved medication appropriateness, though evidence on clinical outcomes remains limited [55].

Prescriber adherence also varied widely, and may influence intervention implementation. GP acceptance rates varied from 27% to 53% in one study [45], while ED physician acceptance was 43% in an RCT using CDSS [34]. In contrast, the SENATOR RCT involving 1537 hospitalised older adults reported lower adherence rates (15%) [59].

Reported factors influencing adherence included perceived clinical relevance, acute care time pressures, prescriber experience and patient-specific factors such as medication preferences.

Stakeholder feedback was reported in only two studies [44, 49], limiting understanding of acceptability and implementation barriers.

### Further research

Further studies, particularly RCTs, should incorporate clinical outcomes and longer-term follow-up. Interventions should prioritise communication pathways and include qualitative methods to identify implementation barriers and improve acceptability among patients, prescribers and pharmacists.

Research should also examine models that support enhanced interprofessional collaboration. Where appropriate, this should include expanded pharmacist roles, such as prescribing authority, which are increasingly being implemented across healthcare systems [60]. Such models may improve the efficiency of implementing agreed recommendations and reduce physician workload.

In addition, future research should incorporate economic evaluations and assess the impact of interventions on patient flow and patient safety.

## Conclusion

Pharmacist-led medication reviews, CDSS and educational/academic detailing were the principal approaches identified to address PIP in older adults in ED and AGU settings. Evidence was heterogeneous and focused mainly on prescribing-related outcomes, with limited assessment of clinical outcomes, prescriber adherence, communication pathways or stakeholder perspectives. These findings highlight opportunities to strengthen medicines optimisation within ED and AGU pathways.

## Supplementary Information

Below is the link to the electronic supplementary material.

Supplementary file1 (PDF 1023 KB)

## Data Availability

No datasets were generated or analysed during the current study.
