# Supplementary material for: Evidence on interventions that target prescribing appropriateness in older adults attending the emergency department and other acute care settings: a scoping review
Source: Int J Clin Pharm. 2026 Jun 18;48(5):1773–86. doi: 10.1007/s11096-026-02170-8 (PMC13569505; doi:10.1007/s11096-026-02170-8)
Supplement: Supplementary file 1 [file 11096_2026_2170_MOESM1_ESM.pdf]

Supplementary Material

Evidence on interventions that target prescribing appropriateness in older adults attending the emergency department and other acute care settings: a scoping review

Authors:

Lauren Fernandes[1], Daniel McCarthy[2], Claire McCormack[2], Aoife Leahy[1], Anne Harnett[2], Abdirahman Mohamed[1], Ahmed Gabr[3], Margaret O'Connor[1,4], Rose Galvin[1,4]

Affiliations:

[1] School of Allied Health, Faculty of Education and Health Sciences, University of Limerick, Limerick, Ireland

[2] School of Medicine, Faculty of Education and Health Sciences, University of Limerick, Limerick, Ireland

[3] University Hospital Limerick, Limerick, Ireland

[4] Ageing Research Centre, Health Research Institute, University of Limerick, Limerick, Ireland

Corresponding author:

Lauren Fernandes (lsarahfernandes@gmail.com or lauren.fernandes@ul.ie)

Table of Contents

Appendix 1 3
- a) Search Terms 3
- b) Detailed Search Strategy for Embase (via Ovid) 4

Appendix 2 6
- a) Basic Study Characteristics 6
- b) Reported Results By Outcome Category 11
- c) Communication Pathways, Prescriber Adherence and Stakeholder Feedback 17

Appendix 3 22
- Preferred Reporting Items for Systematic reviews and Meta-Analyses extension for Scoping Reviews (PRISMA-ScR) Checklist [22] 22

Abbreviations 24

References 25

Appendix 1

a) Search Terms

Research Question: What interventions have been tried to address potentially inappropriate prescribing *(Concept)* among older adults *(Population)* attending the emergency department *(Context (1))* or an acute medical unit *(Context (2))*?				
	Population<br>**Older adults**	Concept<br>**Potentially inappropriate prescribing**	Context (1)<br>**Emergency department**	Context (2)<br>**Acute medical unit**
MeSH Terms	'Aged' OR 'Aged, 80 and Over' OR 'Geriatrics'	'Potentially Inappropriate Medication List' OR 'Inappropriate Prescribing' OR 'Medication Errors'	'Emergency Treatment' OR 'Emergency Medicine' OR 'Emergency Service, Hospital'	
Emtree Terms	'aged' OR 'geriatrics'	'prescribing errors'	'emergency ward' OR 'emergency health service'	'observation unit'
CINAHL Subject Headings	'aged' OR 'aged, 80 and over' OR 'frail elderly'	'inappropriate prescribing' OR 'medication errors'	'emergency medicine' OR 'emergency treatment' OR 'emergency service' OR 'emergency medical service'	'observation units'
Key Words	'aged, 65 and over' OR 'middle aged' OR 'middle-aged' OR 'elder*' OR 'old* adult*' OR 'old* people' OR 'elder* population' OR 'old* population' OR 'elder* people' OR 'elder* adult' OR 'old* patient*' OR 'elder* patient*' OR 'aged population' OR 'ageing population' OR 'aging population' OR 'frail* adult*' OR 'frail* elder*' OR 'geriatric*' OR 'senior citizen*' OR 'senior*' OR 'senium'	'inappropriate prescri*' OR 'inappropriate medic*' OR 'potential* inappropriate prescri*' OR 'potential* inappropriate medic*' OR 'prescri* omission*' OR 'medic* omission*' OR 'drug* omission' OR 'potential* prescri* omission*' OR 'omission* error*' OR 'PIM list*' OR 'medication appropriateness index' OR 'prescri* quality' OR 'prescri* tool*' OR 'suboptimal prescri*' OR 'sub optimal prescri*' OR 'sub-optimal prescri*' OR 'drug prescri*' OR 'Beers criteria' OR 'Stuck AND criteria' OR 'McLeod AND criteria' OR 'Zhan AND criteria' OR 'Beers-Fick criteria' OR 'Rancourt criteria' OR 'HEDIS' OR 'Lindblad criteria' OR 'LaRoche AND criteria' OR 'Japanese Beers criteria' OR 'STOPP version 1' OR 'STOPP criteria' OR 'START criteria' OR 'STOPP START criteria' OR 'Winit-Watjana et al' OR 'Winit-Watjana criteria' OR 'NORGEP' OR 'NORGEP-NH' OR 'Kim AND criteria' OR 'Maio AND criteria' OR 'PRISCUS' OR 'APIT' OR 'Australian prescribing indicators tools' OR 'Mann AND criteria' OR 'Bachyrycz AND criteria' OR 'Chang AND criteria' OR 'Castillo Paramo AND criteria' OR 'Clyne criteria' OR 'Fialova AND criteria' OR 'FORTA AND criteria' OR 'Kunh Thiel AND criteria' OR 'Galan-Retamal AND criteria' OR 'EU 7 PIM list' OR 'Renon-Guiteras AND criteria' OR 'Passi AND criteria' OR 'GheOP3S' OR 'Tommelein AND criteria' OR 'Khodyakov AND criteria' OR 'Mazhar AND criteria' OR 'IFAsPIAM' OR 'STOPPFrail' OR 'LESS-CHRON criteria' OR 'PIM Taiwan criteria' OR 'JAPAN-FORTA' OR 'STOPPFall' OR 'REMEDIeS' OR 'PIP-CPC' OR 'Hong Kong specific PIM list' OR 'Hong Kong specific criteria' OR 'TIME criteria' OR 'PIMDINAC' OR 'Kwanghee Jun AND criteria' OR 'RASP CARDIO list'	'emergency department*' OR 'ED' OR 'A&E' OR 'accident and emergency' OR 'emergency service' OR 'emergency medicine' OR 'emergency medical service*' OR 'hospital emergency service' OR 'emergency hospital service*' OR 'emergency unit*' OR 'emergency ward*' OR 'emergency room*' OR 'trauma center*' OR 'trauma centre*' OR 'trauma unit*' OR 'emergency AND hospital'	'acute medical assessment unit*' OR 'acute medical admission* unit*' OR 'acute assessment unit*' OR 'acute admission* unit' OR 'acute planning unit' OR 'acute medical ward' OR 'acute medical receiving ward' OR 'acute medical care' OR 'combined admissions unit' OR 'combined assessment unit' OR 'clinical decision* unit' OR 'early assessment medical unit' OR 'emergency admission* unit' OR 'immediate care unit' OR 'medical assessment unit' OR 'medical assessment planning unit' OR 'medical emergency department*' OR 'rapid intensive observation' OR 'rapid assessment medical unit' OR 'urgency medicine'

b) Detailed Search Strategy for Embase (via Ovid)

#	Searches	Results
1	(('aged, 65 and over') or 'middle aged' or 'middle-aged' or 'elder*' or 'old* adult*' or 'old* people' or 'elder* population' or 'old* population' or 'elder* people' or 'elder* adult' or 'old* patient*' or 'elder* patient*' or 'aged population' or 'ageing population' or 'aging population' or 'frail* adult*' or 'frail* elder*' or 'geriatric*' or 'senior citizen*' or 'senior*' or 'senium').ab. or (('aged, 65 and over') or 'middle aged' or 'middle-aged' or 'elder*' or 'old* adult*' or 'old* people' or 'elder* population' or 'old* population' or 'elder* people' or 'elder* adult' or 'old* patient*' or 'elder* patient*' or 'aged population' or 'ageing population' or 'aging population' or 'frail* adult*' or 'frail* elder*' or 'geriatric*' or 'senior citizen*' or 'senior*' or 'senium').ti. or ('aged' or 'geriatrics').sh.	4413600
2	((((('emergency department*' or 'ED' or 'A&E' or 'accident) and emergency') or 'emergency service' or 'emergency medicine' or 'emergency medical service*' or 'hospital emergency service' or 'emergency hospital service*' or 'emergency unit*' or 'emergency ward*' or 'emergency room*' or 'trauma center*' or 'trauma centre*' or 'trauma unit*' or 'emergency) and hospital') or 'acute medical assessment unit*' or 'acute medical admission* unit*' or 'acute assessment unit*' or 'acute admission* unit' or 'acute planning unit' or 'acute medical ward' or 'acute medical receiving ward' or 'acute medical care' or 'combined admissions unit' or 'combined assessment unit' or 'clinical decision* unit' or 'early assessment medical unit' or 'emergency admission* unit' or 'immediate care unit' or 'medical assessment unit' or 'medical assessment planning unit' or 'medical emergency department*' or 'rapid intensive observation' or 'rapid assessment medical unit' or 'urgency medicine').ab. or ((((('emergency department*' or 'ED' or 'A&E' or 'accident) and emergency') or 'emergency service' or 'emergency medicine' or 'emergency medical service*' or 'hospital emergency service' or 'emergency hospital service*' or 'emergency unit*' or 'emergency ward*' or 'emergency room*' or 'trauma center*' or 'trauma centre*' or 'trauma unit*' or 'emergency) and hospital') or 'acute medical assessment unit*' or 'acute medical admission* unit*' or 'acute assessment unit*' or 'acute admission* unit' or 'acute planning unit' or 'acute medical ward' or 'acute medical receiving ward' or 'acute medical care' or 'combined admissions unit' or 'combined assessment unit' or 'clinical decision* unit' or 'early assessment medical unit' or 'emergency admission* unit' or 'immediate care unit' or 'medical assessment unit' or 'medical assessment planning unit' or 'medical emergency department*' or 'rapid intensive observation' or 'rapid assessment medical unit' or 'urgency medicine').ti. or ('emergency ward' or 'emergency health service' or 'observation unit').sh.	438855
3	('inappropriate prescri*' or 'inappropriate medic*' or 'potential* inappropriate prescri*' or 'potential* inappropriate medic*' or 'prescri* omission*' or 'medic* omission*' or 'drug* omission' or 'potential* prescri* omission*' or 'omission* error*' or 'PIM list*' or 'medication appropriateness index' or 'prescri* quality' or 'prescri* tool*' or 'suboptimal prescri*' or 'sub optimal prescri*' or 'sub-optimal prescri*' or 'drug prescri*').ab. or (ropriate medic*' or 'prescri* omission*' or 'medic* omission*' or 'drug* omission' or 'potential* prescri* omission*' or 'omission* error*' or 'PIM list*' or 'medication appropriateness index' or 'prescri* quality' or 'prescri* tool*' or 'suboptimal prescri*' or 'sub optimal prescri*' or 'sub-optimal prescri*' or 'drug prescri*').ti. or 'prescribing errors'.sh. or ((('Beers criteria' or 'Stuck) and criteria') or 'McLeod) and criteria') or 'Zhan) and criteria') or 'Beers-Fick criteria' or 'Rancourt criteria' or 'HEDIS' or 'Lindblad criteria' or 'LaRoche) and criteria') or 'Japanese Beers criteria' or 'STOPP version 1' or 'STOPP criteria' or 'START criteria' or 'STOPP START criteria' or 'Winit-Watjana et al' or 'Winit-Watjana criteria' or 'NORGEP' or 'NORGEP-NH' or 'Kim) and criteria') or 'Maio) and criteria') or 'PRISCUS' or 'APIT' or 'Australian prescribing indicators tools' or 'Mann) and criteria') or 'Bachyrycz) and criteria') or 'Chang) and criteria') or 'Castillo Paramo) and criteria') or 'Clyne criteria' or 'Fialova) and criteria') or 'FORTA) and criteria') or 'Kunh Thiel) and criteria') or 'Galan-Retamal) and criteria') or 'EU 7 PIM list' or 'Renon-Guiteras) and criteria') or 'Passi) and criteria') or 'GheOP3S' or 'Tommelein) and criteria') or 'Khodyakov) and criteria') or 'Mazhar) and criteria') or 'IFAsPIAM' or 'STOPPFrail' or 'LESS-CHRON criteria' or 'PIM Taiwan criteria' or 'JAPAN-FORTA' or 'STOPPFall' or 'REMEDIeS' or 'PIP-CPC' or 'Hong Kong specific PIM list' or 'Hong Kong specific criteria' or 'TIME criteria' or 'PIMDINAC' or 'Kwanghee Jun) and criteria') or 'RASP CARDIO list').ab. or	34134

	((('Beers criteria' or 'Stuck) and criteria') or 'McLeod) and criteria') or 'Zhan) and criteria') or 'Beers-Fick criteria' or 'Rancourt criteria' or 'HEDIS' or 'Lindblad criteria' or 'LaRoche) and criteria') or 'Japanese Beers criteria' or 'STOPP version 1' or 'STOPP criteria' or 'START criteria' or 'STOPP START criteria' or 'Winit-Watjana et al' or 'Winit-Watjana criteria' or 'NORGEP' or 'NORGEP-NH' or 'Kim) and criteria') or 'Maio) and criteria') or 'PRISCUS' or 'APIT' or 'Australian prescribing indicators tools' or 'Mann) and criteria') or 'Bachyrycz) and criteria') or 'Chang) and criteria') or 'Castillo Paramo) and criteria') or 'Clyne criteria' or 'Fialova) and criteria') or 'FORTA) and criteria') or 'Kunh Thiel) and criteria') or 'Galan-Retamal) and criteria') or 'EU 7 PIM list' or 'Renon-Guiteras) and criteria') or 'Passi) and criteria') or 'GheOP3S' or 'Tommelein) and criteria') or 'Khodyakov) and criteria') or 'Mazhar) and criteria') or 'IFAsPIAM' or 'STOPPFrail' or 'LESS-CHRON criteria' or 'PIM Taiwan criteria' or 'JAPAN-FORTA' or 'STOPPFall' or 'REMEDIeS' or 'PIP-CPC' or 'Hong Kong specific PIM list' or 'Hong Kong specific criteria' or 'TIME criteria' or 'PIMDINAC' or 'Kwanghee Jun) and criteria') or 'RASP CARDIO list').ti.	
4	1 and 2 and 3	638
5	limit 4 to (abstracts and human and english language and yr="1991 -Current")	603

The search strategy and retrieval numbers shown here reflect the search run on 30 January 2025 through Ovid Embase, via the University of Limerick subscription. A subsequent search update (January – August 2025) was conducted via the Health Service Executive (HSE) Ovid Embase platform due to institutional access changes. Updated retrieval counts are incorporated in the PRISMA flow diagram.

The above search strategy was adapted to other databases including Medline (via Ovid), WebOfScience, Cochrane CENTRAL and CINAHL Ultimate (via EBSCO); as well as to the five databases used in the grey literature search (Proquest, Google Scholar, Bielefeld Academic Search Engine, ICTRP and Clinicaltrials.gov).

For the grey literature databases, advanced search options were not always available, and it was therefore necessary to use more concise search strings.

Appendix 2

a) Basic Study Characteristics

Author<br>Year<br>Country	Study Design	Setting	Participant Characteristics & Number (n)	Outcomes	Study Duration<br>Follow-Up
Stevens<br>2015<br>USA [1]	Before-and-after intervention study (QI initiative)	The ED of a Veterans Affairs (VA) Medical Centre the US	Board-certified physicians (n = 12), a clinical pharmacist (n = 1) & advanced practice providers (n = 2).	• Monthly proportion of PIMs prescribed to older veterans (≥65 years) discharged from the ED	Pre-intervention period = 7 months<br>Post-intervention period = 17 months<br><br>No follow-up
Stevens<br>2017<br>USA [2]	Before-and-after intervention study (QI initiative)	Four VA Medical Centre EDs in the US	Physicians in emergency medicine (n = 22), internal medicine (n = 13), both (n = 1), family practice (n = 2); and advanced practice providers (n = 7).	• Monthly proportion of PIMs prescribed to older veterans (≥65 years) discharged from the ED	Duration – at minimum 6-month pre-intervention period and 12-month post-intervention for each site<br><br>No follow-up
Vaughan<br>2021<br>USA [3]	Before-and-after intervention study (QI initiative)	Three VA Medical Centre EDs in the US	Emergency medicine physicians (n = 283), physician assistants (n = 50), nurse practitioners (n = 29) and pharmacists (n = 6).	• Monthly proportion of PIMs prescribed to older veterans (≥65 years) discharged from the ED	Duration – 6-month pre-intervention period and 12-month post-intervention for each site<br><br>No follow-up
Goldberg<br>2022<br>USA [4]	Quasi-experimental interrupted time series, before-and-after intervention study (QI initiative)	The EDs of two academic hospitals & one community hospital in the US	Emergency medicine physicians (n = 186) and advanced practice providers (n = 61)	• Monthly proportion of PIMs prescribed to older veterans (≥65 years) discharged from the ED	Pre-intervention period = 12 months<br>Post-intervention period = 15 months<br><br>No follow-up

Vaughan<br>2023<br>USA [5]	Parallel cluster-randomised trial (QI initiative)	Eight VA Medical Centre EDs	ED prescribers across the eight sites (n = 1186)	• Monthly proportion of PIMs prescribed to older veterans (≥65 years) discharged from the ED	Duration – 6-month pre-intervention period and 12-month post-intervention period for each site<br><br>No follow-up
Vandenberg<br>2024<br>USA [6]	Prospective before-and-after intervention study (QI initiative)	Four VA Medical Centre EDs	ED prescribers across the four sites (n = 350)	• Monthly proportion of PIMs prescribed to older veterans (≥65 years) discharged from the ED	Duration – 12-month pre-intervention period and 12-month post-intervention period for each site<br>No follow-up
Moss<br>2019<br>USA [7]	Before-and-after intervention study (QI initiative)	The ED of a VA Medical Centre (a tertiary teaching hospital) in the US	Physician residents (63 who received the educational intervention, and 67 who did not) in the VA ED	• Residents' prescribing confidence around PIP in older adults (≥65 years)<br>• PIM prescribing rates	Duration 22 months<br><br>No follow-up
Terrell<br>2009<br>USA [8]	Randomised controlled trial	The ED of a university hospital the US	Emergency medicine resident & faculty physicians<br>(n = 63)	• Proportion of ED visits by older adults (≥65 years) resulting in the prescribing of at least one PIM<br>• Proportion of all medications which were PIMs<br>• Number of times each PIM was initially prescribed<br>• Percentage of times an alternative therapy was chosen by physicians<br>• Reported primary reasons physicians had for rejecting decision support<br>• Physician and patient factors associated with decisions to prescribe PIMs	Duration 30 months<br><br>No follow-up
O'Connor<br>2021<br>UK [9]	Quality improvement study with plan-do-study-act methodology	The clinical decisions unit (CDU) of the ED of a university hospital in the UK	Patients ≥65 years admitted to the CDU (115 in the pre-intervention group & 111 in the post-intervention group)	• Prevalence of PIP, measured using STOPP/START criteria	Pre-intervention period = 2 months<br>Post-intervention period = 1 month<br><br>No follow-up

Spinewine 2007 Belgium [10]	Randomised controlled trial	Acute GEM unit of a university teaching hospital in Belgium	Patients ≥70 years admitted to the acute GEM unit & expected to stay at least 48 hours (n = 203)	• Change in appropriateness of prescribing from admission to discharge, measured using MAI, Beers and ACOVE criteria<br>• Medications taken<br>• Mortality, readmissions, visits to an ED<br>• Satisfaction with information received on medication	Duration 6 months<br><br>Follow-up at 1 month, 3 months and 1 year post-discharge
Lozano-Montoya 2015 Spain [11]	Ambispective, non-randomised study	Acute geriatric unit of the Hospital Universitario Ramón y Cajal in Madrid, Spain	Patients ≥80 years admitted via ED to the acute geriatric unit, with an acute illness (n = 388)	• Outcomes were not defined, however study objective was to determine compliance with STOPP/START recommendations at hospital discharge, and in doing so PIMs and PPOs were measured along with acceptance rates of recommendations (with reasons)	Duration not specified<br><br>No follow-up
Liu 2019 Taiwan [12]	Before-and-after intervention study	The ED of a tertiary medical centre in Southern Taiwan	Patients ≥65 years awaiting hospital admission on basis of ED diagnosis and treatment (243 in preintervention group, and 668 in postintervention group)	• Change in prevalence of hyperpolypharmacy from ED admission to discharge<br>• Change in prevalence of PIP from admission to discharge	Preintervention period = 3 months, postintervention period = 5 months<br><br>No follow-up
Santolaya-Perrin 2019 Spain [13]	Randomised controlled trial	The EDs of four Spanish hospitals	Patients ≥65 years (taking at least one chronic medication) presenting to the ED (342 in control group, 323 in intervention group)	• Rate of ED visits and hospital admissions per patient-year, measured at 3, 6 and 12 months	Duration 8 months<br><br>Follow-up for 12 months
Gutiérrez-Valencia 2019 Spain [14]	Prospective before-and-after study	Acute geriatric unit (AGU) in a tertiary public hospital in Spain	Patients ≥75 years admitted to an acute geriatric unit (n = 234)	• Change in prevalence of polypharmacy and hyperpolypharmacy from admission to discharge<br>• Change in mean number of medications<br>• Change in prevalence of PIP per STOPP/START criteria from admission to discharge<br>• DRP detected by pharmacist during medication review at admission, categorised by type	Duration 6 months<br><br>No follow-up
Houlind 2020	Longitudinal feasibility study	The ED of a university hospital in Denmark	Patients ≥65 years on ≥5 long-term medications admitted to	• Change in MAI from admission to hospital till 30 days post-discharge<br>• Change in AOU scores	Duration 9 months

Denmark [15]			the medical ED (n = 60, with 39 completing follow-up at 30 days post-discharge)	• Proportion of patients where intervention was completed pre-discharge without affecting hospital LOS<br>• Frequency of geriatrician agreeing with pharmacist recommendations<br>• Frequency of implementation of pharmacist recommendations by geriatrician	Follow-up at 30 days post-discharge
Farhat 2022 Switzerland [16]	Randomised controlled trial	Acute Care for Elders (ACE) unit of a university hospital in Switzerland	Patients ≥65 years (with at least one geriatric syndrome and an acute illness/exacerbated chronic condition) admitted to the ACE unit (n = 123)	• Prevalence & number of PIP<br>• Number & acceptance rate of interventions<br>• Number of prescribed medications<br>• Falls, ADL scores, delirium, LOS, short- and long-term rehospitalisation	Duration 14 months<br><br>Follow-up at 1 month and 3 months post-discharge
Jovevski 2023 USA [17]	Retrospective before-and-after intervention pilot study	An urban Veterans Affairs ED in the US	Patients ≥75 years presenting to ED who had a positive ISAR score and were discharged home (149 patients each in the pre- and post-intervention groups)	• Per-medication deprescribing rate<br>• 30-day primary care practitioner visits<br>• 7-day and 30-day ED visits<br>• 7-day and 30-day hospitalisations<br>• 60-day mortality	Duration 12 months each for pre- and post-intervention periods<br><br>Follow-up at 7, 30 and 60 days post-discharge
Atey 2023 Australia [18]	Pragmatic concurrent controlled study	The ED of a referral public & teaching hospital	Patients ≥65 years presenting to ED & subsequently admitted to an acute medical unit, taking ≥1 regular medication (n = 321, with 107 per intervention group)	• Percentage of patients prescribed ≥1 PIM at ED discharge<br>• ≥1 PIM at hospital discharge<br>• Median number of PIMs per patient and per study group<br>• Relative risk reduction in using ≥1 PIM in PPMC vs BPMH group	Duration 12 months<br><br>No follow-up
Reilly 2024 Australia [19]	Mixed methods	The ED of a major tertiary referral hospital	Patients ≥70 years with ≥3 comorbidities, taking ≥5 medications daily, who were discharged from the ED (n = 1214 received the intervention, n = 35 in follow-up study)	• Number of drugs<br>• PIMs identified by STOPP criteria<br>• PPOs identified by START criteria<br>• Proportion of recommendations accepted by GP<br>• Patient satisfaction with pharmacist-led service	Duration 12 months<br><br>Follow-up at least 28 days post-intervention

Elder<br>2025<br>USA<br>[20]	Multicentre, retrospective pre-post implementation study	Nineteen EDs in the US	Patients ≥65 years who were prescribed a PIM either during their ED visit or at ED discharge (2982 & 2832 ED encounters; 721 & 588 ED discharge encoun-ters from pre- and post-implementation periods respectively)	• Proportion of targeted PIMs adherent to CDS recommendations for ED orders<br>• Proportion of targeted PIMs adherent to CDS recommendations for discharge prescriptions<br>• Proportion of all targeted PIMs ordered through CDS order sets, in post-implementation group<br>• Proportion of targeted PIMs adherent to CDS recommendations when ordered through CDS order sets	Duration 2 months each for pre- and post-intervention periods<br><br>No follow-up
Hellinger<br>2025<br>Germany<br>[21]	Consecutive, controlled intervention study	The ED of a tertiary university hospital	Patients ≥65 years presenting to an ED after a fall which had occurred within the preceding 24 hours	Outcomes not explicitly mentioned, but the following were measured in the routine vs intervention groups:<br>• Number of patients prescribed at least one FRID at ED discharge<br>• Number of FRIDs at ED discharge<br>• Number of drug therapy modifications	Duration 3 months each for pre-and post-intervention periods<br><br>No follow-up

b) Reported Results By Outcome Category

Author Year Country	Prevalence of Potentially Inappropriate Prescribing Among ED Providers	Prevalence of Potentially Inappropriate Chronic Medications	Number of Medications (Prevalence of Polypharmacy)	Clinical Outcomes
Stevens 2015 USA [1]	Average monthly PIM prescribing rates improved from 9.4% (1.5) in the pre-implementation period, to 4.6% (1.0) (RR = 0.48, 95% CI 0.40-0.59, p<0.001) in the post-implementation period.			
Stevens 2017 USA [2]	Average monthly PIM prescribing rate by site:<br>Site 1: 11.9% (1.8) pre-implementation to 5.1% (1.5) post-implementation (p<0.0001)<br>Site 2: 8.2% (0.8) pre-implementation to 4.5% (1.0) post-implementation (p<0.0001)<br>Site 3: 8.9% (1.9) pre-implementation to 6.1% (1.7) post-implementation (p=0.0007)<br>Site 4: 7.4% (1.7) pre-implementation to 5.7% (0.8) post-implementation (p=0.04)			
Vaughan 2021 USA [3]	Average monthly PIM prescribing rate by site:<br>Site 1: 5.6% (95% CI = 5.0–6.3) pre-implementation to 5.1% (95% CI = 4.7–5.5) post-implementation (p<0.02)<br>Site 2: 5.8% (95% CI = 5.0–6.6) pre-implementation to 5.4% (95% CI = 4.8–6.0) post-implementation (p=0.62)<br>Site 3: 7.3% (95% CI = 6.4–9.2) pre-implementation to 7.5% (95% CI = 6.6–8.4) post-implementation (p=0.64)			
Goldberg 2022 USA [4]	Average monthly PIM prescribing rates improved from 9.3% (95% CI = 8.82–9.78) pre-implementation to 8.62% (95% CI = 8.14–9.1) post-implementation (p<0.01)			

Vaughan<br>2023<br>USA [5]	Average monthly PIM prescribing rates improved significantly from 8.00% pre-implementation to 7.07% post-implementation (p=0.0006) in the four sites using the academic detailing approach to feedback, while a nonsignificant increase from 8.04% pre-implementation to 8.10% post-implementation for the four sites using the dashboard approach to feedback. Two of the four academic detailing sites and one of the four dashboard sites showed a significant reduction in PIM prescribing rates (figures not provided except for the latter, which was the site showing the largest reduction – from 10.49% at baseline to 5.8% post-implementation (p≤0.001)).<br>The odds of a PIM being prescribed at discharge at 12 months post-implementation were 14% higher in the dashboard sites than the academic detailing sites (OR 1.14, 95% CI 1.08-1.22)			
Vandenberg<br>2024<br>USA [6]	Average monthly PIM prescribing rate by site:<br>Hub site: 8.9% (95% CI = 8.1–9.6) pre-implementation to 3.6% (95% CI = 3.6–9.6) post-implementation (p<0.001)<br>Spoke site 1: 12.2% (95% CI = 11.2–13.2) pre-implementation to 7.13% (95% CI = 6.1–8.1) post-implementation (p<0.001)<br>Spoke site 2: 11.3% (95% CI = 10.1–12.6) pre-implementation to 7.5% (95% CI = 6.4–8.8) post-implementation (p=0.045)<br>Spoke site 3: 16.2% (95% CI = 14.9–17.4) pre-implementation to 11.7% (95% CI = 10.3–13.1) post-implementation (p<0.001)			
Moss<br>2019<br>USA [7]	The 63 resident physicians who received an educational intervention to address PIP had significantly lower PIM prescribing rates than their 67 peers who had not, with a rate			

	ratio of 0.73 (95% CI 0.632-0.850; p<0.0001).			
Terrell 2009 USA [8]	The proportion of ED visits by older adults resulting in the prescribing of ≥1 PIM was only 2.6% in the intervention group, vs 3.9% in the control group (p=0.02, OR = 0.55 with 95% CI 0.4-2.3%). The proportion of all medications prescribed which were PIMs also decreased significantly from 5.4% to 3.4% (p=0.006, OR = 0.59 with 95% CI 0.41-0.85).			
O'Connor 2021 UK [9]	A significant reduction was noted in the number of patients who had ≥1 prescribing error, from 37% in the pre-intervention group to 26% in the post-intervention group (p=0.04). The reduction in those with ≥1 START error was insignificant (24.6% to 20.7%, p=0.24), while the proportion of patients with ≥1 STOPP error was significantly reduced (17.5% to 9.0%, p=0.03).			
Spinewine 2007 Belgium [10]		Patients in the intervention group had significantly greater improvements in PIP measured according to the MAI and ACOVE criteria from admission to discharge than those in the control group (OR 6.1, 95% CI 2.2-17.0 & OR 9.1, 95% CI 4.2-21.6), while improvements in PIP measured per the Beers criteria were similar (OR 0.6, 95% CI 0.3-1.1).		There were no statistically significant differences between the intervention and control groups with regards to rates of death (22.5% vs 30.1%, p=0.30) and emergency visits (7.9% vs 12.0%, p=0.45) at one-year post-discharge. Readmission rates were also similar between groups (32.6% vs 33.7%, p=1.0).
Lozano-Montoya 2015 Spain [11]		The proportion of PIMs identified per the STOPP criteria which were discontinued was 87.0% (247 of 284), while only 33.5% of potential prescribing omissions (PPOs) identified according to the START criteria were commenced.	A decrease was noted in the mean number of medications prescribed from admission to discharge (8.1 (3.7) to 7.0 (3.3)), although it was not specified whether this was statistically significant.	

Liu 2019 Taiwan [12]		There was a significant improvement in the number of PIMs that were successfully reduced in the post-intervention group when compared to the pre-intervention group (68.4% vs 49.1%, p<0.001).	Rates of successful reduction in hyperpolypharmacy were higher post-intervention (79.4% vs 65.3%, p<0.001), with a decrease in rates of hyperpolypharmacy from 70% to 59.7% and mean number of medications from 12.5 (2.7) to 6.9 (3.0) (in patients with hyperpolypharmacy).	
Santolaya-Perrin 2019 Spain [13]				No statistically significant reduction in rate of hospital admissions and emergency visits between the intervention & control groups at 3, 6 or 12 months (rate ratios 0.808 with 95% CI 0.617 to 1.059, 0.888 with 95% CI 0.696 to 1.134 and 0.954 with 95% CI 0.772 to 1.179). Statistically significant reductions only noted at 3 months in the two sites where GP acceptance rates were highest (0.452, 95% CI 0.222-0.923 & 0.567, 95% CI 0.328-0.983).
Gutiérrez-Valencia 2019 Spain [14]		There were significant reductions in the rates of PIP measured using STOPP criteria (−19.2%, 95% CI −24.9, −13.6, p<0.001) as well as by START criteria (−6.8%, 95% CI −10.1, −3.5, p<0.001), with reductions from 184 to 139 patients and 151 to 135 patients with STOPP and START criteria respectively.	Significant improvements from admission to discharge in terms of rates of polypharmacy (−10.2%, 95% CI −15.3, −5.2, p<0.001), rates of hyperpolypharmacy (−16.6%, 95% CI −22.3 −11.0, p<0.001), and reductions in the number of medications per patient (−1.4, 95% CI −1.8, −1.0, p<0.001).	
Houlind 2020 Denmark [15]		There was a significant reduction in median MAI scores from admission to post-intervention & 30 days post-discharge (14 with IQR 8-20 to 6 with IQR 4-10 and 8 with IQR 2-13, p<0.001) as well as in the number of	One or more medications were deprescribed for 72% (n = 28) of patients, with 1.6 medications deprescribed per patient on average. While there was a significant reduction in the	

		patients with an AOU score of ≥1 (36% to 8% and 10%, p<0.001). Of the changes made during the intervention on admission, 83% were sustained at 30 days post-discharge.	number of medications at admission vs 30 days post-discharge (11.9 (3.2) vs 11.3 (3.0), p=0.038), there was no significant difference between admission and 30 days post-discharge (p=0.36).	
Farhat 2022 Switzerland [16]		A significant decrease in PIP was demonstrated in both the STOPP/START (34%) and PIM-check (31%) arms (p value not provided), but with no significant difference between arms (p=0.825). There was also no difference between groups in the mean decrease in number of PIPs per patient (p=0.954). Central nervous system drug deprescribing was significantly higher in the STOPP/START arm (p=0.025).	There was a higher prevalence of hyperpolypharmacy at discharge in the PIM-Check arm than the STOPP/START arm (23.3% vs 8.1%, p=0.049). There was no significant difference between groups in the number of drugs prescribed between admission and discharge (p=0.252).	ADL scores at discharge were higher in the STOPP/START than PIM-Check arm (4.42 vs 3.77, p=0.040), predicted by use of STOPP/START, CNS drug deprescribing & shorter LOS. Mean ADL scores decreased more in the PIM-Check arm (-1.13 vs -0.29, p=0.001). No changes were found between groups in incidence of falls (p>0.999), delirium (p=0.68), mean LOS (p=0.48) or readmission at 1 month or 3 months (p=0.35, p=0.8).
Jovevski 2023 USA [17]		The PIM deprescribing case rate at 60 days increased significantly from 11.1% pre-intervention to 57.1% post-intervention (p<0.001), with 91% of PIMs remaining unchanged pre-intervention vs only 49% post-intervention (p<0.05).		There were no significant changes at 7- or 30-day follow-up in rates of ED visits (p=0.6814 & p=0.3598) or hospitalization (p>0.9999 & p=0.4116), and mortality rates at 60 days also remained unchanged (p>0.9999).
Atey 2023 Australia [18]		The risk of being prescribed ≥1 PIM at ED departure decreased by 14.6% (95% CI 12-4-17.8%) and 19.6% (95% CI 16.8-23.7%) in the PPMC group vs the early BPMH and usual care groups respectively (p=0.040), with no significant differences at hospital discharge (p=0.27). The median number of PIMs per patient was lower in the PPMC group than the other two groups at ED departure (p=0.046), with no significant change		

		at hospital discharge (p=0.54). Significant improvements were noted in the percentage of patients prescribed ≥1 PIM at ED departure (p=0.040) within the PPMC group, but not in the BPMH or usual care groups (p=0.88, p>0.99). None of the study groups showed a significant change in risk of being prescribed ≥1 PIM or the median per-patient PIM rate at hospital discharge.		
Reilly 2024 Australia [19]		At follow-up, 83% (25/30) patients had at least one STOPP recommendation followed by their GP, while 69% (9/13) patients had at least one START recommendation followed.		
Elder 2025 USA [20]		The proportion of targeted PIMs adherent to CDS recommendations was higher in the post-implementation group for ED orders (71% vs 52%, 95% CI 16.8-21.3%) as well as discharge prescriptions (31.7% vs 0.5%, 95% CI 27.5-34.7%).		
Hellinger 2025 Germany [21]		There were 85 vs 68 patients with at least one FRID prescribed at ED discharge in the routine vs pharmaceutical care groups (p = 0.010). The median number of FRIDs prescribed at ED discharge also reduced significantly between the routine and pharmaceutical care groups, from 2 to 1 (p = 0.003).		

c) Communication Pathways, Prescriber Adherence and Stakeholder Feedback

Author Year Country	Communication Pathways	Prescriber Adherence	Stakeholder Feedback
Stevens 2015 USA [1]	Education was provided to ED prescribers by way of geriatric pharmacology lectures presented by a geriatrician and a review of the 2012 Beers Criteria Update during a journal club. Reminder cards showing the most frequently prescribed PIMs/ PIM categories were displayed at ED computer stations. Point-of-prescribing education was also provided via informatics-based clinical decision support, along with suggested dose adjustments per age and renal function. Finally, prescribers received individual feedback on their prescribing patterns from an ED physician, which was then peer benchmarked.		
Stevens 2017 USA [2]	Education was provided to ED prescribers by way of lectures by geriatricians and/or clinical pharmacists, as well as via one-on-one academic detailing feedback sessions tailored to individual prescribing patterns. Each ED provider also received monthly reports including the proportion of total medications prescribed in the ED that were PIMs, a breakdown of PIM drug classes and peer benchmarking for each month. Informatics-based point-of-prescribing education was also provided regarding which medications were deemed inappropriate for older adults.		
Vaughan 2021 USA [3]	Education was provided to ED prescribers by an on-site EQUIPPED champion. Individualised academic detailing was carried out with provider audit & feedback and peer benchmarking. Informatics-based point-of-prescribing education was also provided as previously described.		
Goldberg 2022 USA [4]	Geriatric prescribing teaching was provided during grand rounds, and reminder cards listing the most frequent PIMs were displayed at ED computer stations. Academic detailing and feedback were carried out as per the above studies, as was point-of-prescribing education.		
Vaughan 2023 USA [5]	For the four sites using the academic detailing approach, a local EQUIPPED champion provided education to ED prescribers using individual academic detailing – with additional academic one-on-one sessions for providers who had higher rates of PIM prescribing		

	than the mean rate for their site that month. The local EQUIPPED champion answered questions that arose and engaged ED providers during sessions regarding factors that might impact prescribing behaviour at their site. For the four sites using the dashboard approach, general information was provided on the EQUIPPED intervention and the Beers Criteria, monthly individual prescribing reports were emailed to providers, peer benchmarking data were sent via the PIM dashboard (providers notified of same via email) and providers could choose to notify ED leadership/site champions regarding factors that might affect prescribing behaviour.		
Vandenberg 2024 USA [6]	A clinical decision support package was provided, comprising of didactic education, point-of-prescribing advice via EMR-embedded order sets, and audit & feedback delivered by a local clinical champion. Three of the four sites (known as the 'spoke' sites in the hub-and-spoke approach to implementation) received order set updates, education and audit and feedback reports from the fourth site, known as the 'hub'.		
Moss 2019 USA [7]	Education was provided to resident physicians as a group at the start of their emergency medicine (EM) rotation. The first academic detailing session was a lecture on geriatric prescribing and pharmacology delivered by an EQUIPPED geriatrician/ clinical pharmacist pair. Informatics-based point-of-prescribing education was also made available to residents. Halfway through their 4-week EM rotation, individual reports on PIM prescribing rates were provided to residents. Computer cards identifying the most frequently prescribed PIMs were also used.		
Terrell 2009 USA [8]	Computerised clinical decision support was provided to physicians in the intervention group when attempting to prescribe one of a subset of targeted PIMs to an older adult being discharged from the ED, and in these instances a recommendation was provided for a safer alternative medication which could either be accepted or rejected. When the recommendation was rejected, physicians were asked to select a reason from a list of options.	A total of 49 out of 114 decision support recommendations (43%) were accepted by physicians. The commonest reasons for rejection were that the patient had previously tolerated the medication (40%), that it was a medication refill (17%), that there was no good substitute (16%), that the dose had been lowered (16%) or patient preference (6%).	
O'Connor 2021	Awareness was raised around the most common prescribing errors (comprising 8 STOPP errors and 11 START errors) in a Clinical		

UK [9]	Decisions Unit over one month, by way of emails, presentations and posters targeting prescribers.		
Spinewine 2007 Belgium [10]	A pharmacist took a medication history and prepared a pharmaceutical care plan for each patient. They then discussed each opportunity for optimisation with the prescriber (with each intervention then either accepted or rejected) and answered all questions regarding medications. Written and oral information was provided on discharge to both the patient/caregiver and the GP.		Satisfaction with information received on medications was measured as a secondary outcome at one-month post-discharge. A higher proportion of patients were satisfied in the intervention group than in the control group (80.0% vs 60.9%), but this difference was not statistically significant (p=0.1).
Lozano-Montoya 2015 Spain [11]	A clinical pharmacist took a medication history from each patient and performed a medication review to detect PIMs. Findings were discussed with the geriatric multidisciplinary team and a consensus was reached regarding recommendations. The pharmacist would then inform the patient and caregiver about the recommended discharge medications and provide written information.	Only 37 of 284 of STOPP recommendations (23%) were rejected, mostly due to other therapeutic needs being prioritised (lorazepam, n=12, risperidone, n=5, other, n=18). Most START recommendations (264/397 i.e. 66.5%) were rejected. with the most frequent reasons cited as severe disability (n=90), alternative effective treatments (n=38) and risk of severe adverse effects (n=32).	
Liu 2019 Taiwan [12]	A computer platform used integrated medication lists to identify patients with hyperpolypharmacy or PIMs and transmit this information to the clinical pharmacist, who would then display treatment recommendations to all treating physicians in the Hospital Information System. Physicians could accept or reject recommendations, and in instances of rejection were contacted by the pharmacist.		
Santolaya-Perrin 2019 Spain [13]	A pharmacist performed a medication history and review for each patient to identify PIPs with subsequent discussion with the study emergency specialist, who would deem PIPs either correctly identified or not. Recommendations were emailed to the GP, entered into an electronic clinical record or mentioned in a discharge report addressed to the GP.	One-month after recommendations were made, clinical records were reviewed to assess adherence. Overall rates of GP acceptance of recommendations: Site 1 – 27%, Site 2 – 31%, Site 3 – 52% and Site 4 – 53%.	
Gutiérrez-Valencia 2019 Spain [14]	A research pharmacist took a medication history followed by a CDSS-assisted medication review and then shared a written pharmaceutical care plan in the electronic patient record (accompanied by verbal communication when necessary). The pharmacist also answered questions about medications from patients or healthcare professionals on demand.		

Houlind 2020 Denmark [15]	A clinical pharmacist performed a medicines reconciliation on admission, followed by a medication review upon which basis deprescribing recommendations were made and discussed with the ED-based geriatrician, who would then accept, reject or alter the recommendation and document their decisions in the patient's electronic record. Accepted recommendations were either implemented in the ED or communicated to another hospital ward or the patient's GP. On discharge from hospital, each patient received a copy of their updated medication list.		
Farhat 2022 Switzerland [16]	A research pharmacist collected each patient's medication list and performed a medication review. Recommendations made during this review were then either accepted or rejected by the attending physician, with reasons documented and changes to regular medications communicated to the outpatient physician.	Physician acceptance rate for interventions in the PIM-Check and STOPP/START arms of the study were 32% (n=43/135) and 43% (n=22/51) respectively.	
Jovevski 2023 USA [17]	Nursing staff conducted ISAR scores for adults ≥75 attending the ED and documented them in the EHR. Computer-generated alerts were sent to the clinical pharmacist for ISAR-positive patients, prompting a pharmacist-led medication review which aimed to identify PIMs. The identified PIMs along with proposed recommendations were documented in the EHR, which would send an alert to the PCP & require them to cosign and acknowledge that they had seen the recommendations.		The ED provider champions designated to educate PCPs reported that they had a very positive response to the idea of a deprescribing intervention (although no formal surveys were performed).
Reilly 2024 Australia [19]	Pharmacist recommendations were communicated electronically to the patient's GP post-discharge from ED.<br><br>Patients were given an updated list of medications on ED discharge, along with a letter for their GP containing recommendations.	At follow-up, in 26% (9/35) of cases, no recommendations were actioned by the GP.<br><br>Reasons provided were that the patient was felt to be stable on their usual regimen, or that the relevant hospital specialist was reluctant to change their current medications.	Participants were satisfied with ED pharmacist involvement and information provided, with respective mean Likert scores of 4.97/5 and 4.95/5. Only 4/45 GPs provided feedback when contacted. Three had positive feedback while one felt recommendations to be inappropriate in the context of their patient's detailed history.
Atey 2023 Australia [18]	In one intervention arm of the study a best possible medication history (BPMH) was performed by a pharmacist and made available to the medical officer (without any discussion taking place). The medical officer would then chart the medications. In the PPMC (partnered pharmacist medication charting) intervention arm, the BPMH was followed by the formation of treatment plan made in collaboration between the medical officer		

	and pharmacist. No mention made of communication of the treatment plan to the patient, caregiver or PCP.		
Elder 2025 USA [20]	Clinical decision support was provided to physicians in the post-implementation group by way of order panels when attempting to prescribe a targeted PIMs to an older adult either during their ED encounter or when being discharged from the ED. No mention made of communication of the treatment plan to the patient, caregiver or PCP.	The proportion of all targeted PIMs ordered through CDS order sets in the post-implementation group was 62.1%, and 90% of these orders were adherent to CDS order panels. For ED discharge prescriptions in the post-implementation group, 36.7% of targeted PIMs were ordered using CDS panels, and of these 80.4% were adherent to recommendations.	
Hellinger 2025 Germany [21]	A clinical pharmacist working in the ED would identify FRIDs and then discuss potential drug therapy modifications with the treating physician. The joint plan would then be documented in discharge notes, and either communicated to the ward (in the event of hospital admission) or given to patients to discuss with their GP.	The number of drug therapy modifications made among patients taking at least one FRID was seven (out of 78 patients) in the routine group vs 83 (out of 89) in the pharmaceutical care group.	

Appendix 3

Preferred Reporting Items for Systematic reviews and Meta-Analyses extension for Scoping Reviews (PRISMA-ScR) Checklist [22]

SECTION	ITEM	PRISMA-ScR CHECKLIST ITEM	REPORTED ON PAGE #
**TITLE**			
Title	1	Identify the report as a scoping review.	1
**ABSTRACT**			
Structured summary	2	Provide a structured summary that includes (as applicable): background, objectives, eligibility criteria, sources of evidence, charting methods, results, and conclusions that relate to the review questions and objectives.	2-3
**INTRODUCTION**			
Rationale	3	Describe the rationale for the review in the context of what is already known. Explain why the review questions/objectives lend themselves to a scoping review approach.	6-7
Objectives	4	Provide an explicit statement of the questions and objectives being addressed with reference to their key elements (e.g., population or participants, concepts, and context) or other relevant key elements used to conceptualize the review questions and/or objectives.	7
**METHODS**			
Protocol and registration	5	Indicate whether a review protocol exists; state if and where it can be accessed (e.g., a Web address); and if available, provide registration information, including the registration number.	7
Eligibility criteria	6	Specify characteristics of the sources of evidence used as eligibility criteria (e.g., years considered, language, and publication status), and provide a rationale.	8
Information sources*	7	Describe all information sources in the search (e.g., databases with dates of coverage and contact with authors to identify additional sources), as well as the date the most recent search was executed.	7-8
Search	8	Present the full electronic search strategy for at least 1 database, including any limits used, such that it could be repeated.	Appendix 1b
Selection of sources of evidence†	9	State the process for selecting sources of evidence (i.e., screening and eligibility) included in the scoping review.	9
Data charting process‡	10	Describe the methods of charting data from the included sources of evidence (e.g., calibrated forms or forms that have been tested by the team before their use, and whether data charting was done independently or in duplicate) and any processes for obtaining and confirming data from investigators.	9
Data items	11	List and define all variables for which data were sought and any assumptions and simplifications made.	9
Critical appraisal of individual sources of evidence§	12	If done, provide a rationale for conducting a critical appraisal of included sources of evidence; describe the methods used and how this information was used in any data synthesis (if appropriate).	N/A

SECTION	ITEM	PRISMA-ScR CHECKLIST ITEM	REPORTED ON PAGE #
Synthesis of results	13	Describe the methods of handling and summarizing the data that were charted.	9
**RESULTS**			
Selection of sources of evidence	14	Give numbers of sources of evidence screened, assessed for eligibility, and included in the review, with reasons for exclusions at each stage, ideally using a flow diagram.	10
Characteristics of sources of evidence	15	For each source of evidence, present characteristics for which data were charted and provide the citations.	11-19 and Appendix 2
Critical appraisal within sources of evidence	16	If done, present data on critical appraisal of included sources of evidence (see item 12).	N/A
Results of individual sources of evidence	17	For each included source of evidence, present the relevant data that were charted that relate to the review questions and objectives.	11-19 and Appendix 2
Synthesis of results	18	Summarize and/or present the charting results as they relate to the review questions and objectives.	11-19
**DISCUSSION**			
Summary of evidence	19	Summarize the main results (including an overview of concepts, themes, and types of evidence available), link to the review questions and objectives, and consider the relevance to key groups.	20-23
Limitations	20	Discuss the limitations of the scoping review process.	20
Conclusions	21	Provide a general interpretation of the results with respect to the review questions and objectives, as well as potential implications and/or next steps.	23
**FUNDING**			
Funding	22	Describe sources of funding for the included sources of evidence, as well as sources of funding for the scoping review. Describe the role of the funders of the scoping review.	29

JBI = Joanna Briggs Institute; PRISMA-ScR = Preferred Reporting Items for Systematic reviews and Meta-Analyses extension for Scoping Reviews.
* Where *sources of evidence* (see second footnote) are compiled from, such as bibliographic databases, social media platforms, and Web sites.
† A more inclusive/heterogeneous term used to account for the different types of evidence or data sources (e.g., quantitative and/or qualitative research, expert opinion, and policy documents) that may be eligible in a scoping review as opposed to only studies. This is not to be confused with *information sources* (see first footnote).
‡ The frameworks by Arksey and O'Malley (6) and Levac and colleagues (7) and the JBI guidance (4, 5) refer to the process of data extraction in a scoping review as data charting.
§ The process of systematically examining research evidence to assess its validity, results, and relevance before using it to inform a decision. This term is used for items 12 and 19 instead of "risk of bias" (which is more applicable to systematic reviews of interventions) to include and acknowledge the various sources of evidence that may be used in a scoping review (e.g., quantitative and/or qualitative research, expert opinion, and policy document).

From: Tricco AC, Lillie E, Zarin W, O'Brien KK, Colquhoun H, Levac D, et al. PRISMA Extension for Scoping Reviews (PRISMAScR): Checklist and Explanation. Ann Intern Med. 2018;169:467–473. doi: 10.7326/M18-0850.

Abbreviations

ACE unit = acute care of the elderly unit

ACOVE underuse criteria = Assessing Care of Vulnerable Elders underuse criteria

ADL = activity of daily living

ADR = adverse drug reaction

AGU = acute geriatric unit

AOU index = Assessment of Underutilisation index

BPMH = best possible medication history

CDSS = clinical decision support systems

CDU = clinical decisions unit

CGA = comprehensive geriatric assessment

CI = confidence interval

DRP = drug-related problem

ED = emergency department

EHR = electronic health record

EM = emergency medicine

EMR = electronic medical record

EQUIPPED = Enhancing Quality of Prescribing Practices for Older Veterans Discharged from the Emergency Department

FRID = Falls Risk Increasing DrugGEM unit = geriatric emergency medicine unit

GP = general practitioner

IQR = interquartile range

ISAR = Identification of Seniors At Risk

JBI = Joanna Briggs Institute

LOS = length of stay

MAI = Medication Appropriateness Index

OR = odds ratio

PCP = primary care practitioner

PIM = potentially inappropriate medication

PIP = potentially inappropriate prescribing

PPMC = partnered pharmacist medication charting

PPO = potential prescribing omission

PRISMA = Preferred Reporting Items for Systematic Reviews and Meta-Analyses

QI = quality improvement

RCT = randomised controlled trial

RR = risk/rate ratio

STOPP/START = Screening Tool of Older Persons Prescriptions/Screening Tool to Alert Doctors to Right Treatment

VA = Veterans Affairs

References

1. Stevens MB, Hastings SN, Powers J, et al. Enhancing the quality of prescribing practices for older veterans discharged from the emergency department (EQUiPPED): Preliminary results from enhancing quality of prescribing practices for older veterans discharged from the emergency department, a novel multicomponent interdisciplinary quality improvement initiative. J Am Geriatr Soc. 2015;63(5):1025-9. https://doi.org/10.1111/jgs.13404.
2. Stevens M, Hastings SN, Markland AD, et al. Enhancing Quality of Provider Practices for Older Adults in the Emergency Department (EQUiPPED). J Am Geriatr Soc. 2017;65(7):1609-14. https://doi.org/10.1111/jgs.14890.
3. Vaughan CP, Hwang U, Vandenberg AE, et al. Early prescribing outcomes after exporting the EQUIPPED medication safety improvement programme. BMJ Open Qual. 2021;10(4):e001369. https://doi.org/10.1136/bmjoq-2021-001369.
4. Goldberg EM, Lin TR, Cunha CB, et al. Enhancing the quality of prescribing practices for older adults discharged from the emergency department in Rhode Island. J Am Geriatr Soc. 2022;70(10):2905-14. https://doi.org/10.1111/jgs.17955.
5. Vaughan CP, Burningham Z, Kelleher JL, et al. A cluster-randomized trial of two implementation strategies to deliver audit and feedback in the EQUIPPED medication safety program. Acad Emerg Med. 2023;30(4):340-8. https://doi.org/10.1111/acem.14697.
6. Vandenberg AE, Hwang U, Das S, et al. Scaling the EQUIPPED medication safety program: Traditional and hub-and-spoke implementation models. J Am Geriatr Soc. 2024;72(7):2184-94. https://doi.org/10.1111/jgs.18746.
7. Moss JM, Bryan WE, Wilkerson LM, et al. An Interdisciplinary Academic Detailing Approach to Decrease Inappropriate Medication Prescribing by Physician Residents for Older Veterans Treated in the Emergency Department. J Pharm Pract. 2019;32(2):167-74. https://doi.org/10.1177/0897190017747424.
8. Terrell KM, Perkins AJ, Dexter PR, et al. Computerized decision support to reduce potentially inappropriate prescribing to older emergency department patients: A randomized, controlled trial. J Am Geriatr Soc. 2009;57(8):1388-94. https://doi.org/10.1111/j.1532-5415.2009.02352.x.
9. O'Connor J, Adabavazeh B, Choi H, et al. Use of the STOPP and START criteria to address polypharmacy for elderly patients in University Hospital Lewisham Clinical Decisions Unit. Hong Kong J Emerg Med. 2021;28(2):79-84. https://doi.org/10.1177/1024907919849358.
10. Spinewine A, Swine C, Dhillon S, et al. Effect of a collaborative approach on the quality of prescribing for geriatric inpatients: a randomized, controlled trial. J Am Geriatr Soc. 2007;55(5):658-65. https://doi.org/10.1111/j.1532-5415.2007.01132.x.
11. Lozano-Montoya I, Vélez-Diaz-Pallarés M, Delgado-Silveira E, et al. Potentially inappropriate prescribing detected by STOPP-START criteria: are they really inappropriate? Age Ageing. 2015;44(5):861-6. https://doi.org/10.1093/ageing/afv079.
12. Liu YL, Chu LL, Su HC, et al. Impact of Computer-Based and Pharmacist-Assisted Medication Review Initiated in the Emergency Department. J Am Geriatr Soc. 2019;67(11):2298-304. https://doi.org/10.1111/jgs.16078.
13. Santolaya-Perrin R, Calderon-Hernanz B, Jimenez-Diaz G, et al. The efficacy of a medication review programme conducted in an emergency department. Int J Clin Pharm. 2019;41(3):757-66. https://doi.org/10.1007/s11096-019-00836-0.
14. Gutiérrez-Valencia M, Izquierdo M, Beobide-Telleria I, et al. Medicine optimization strategy in an acute geriatric unit: The pharmacist in the geriatric team. Geriatr Gerontol Int. 2019;19(6):530-6. https://doi.org/10.1111/ggi.13659.

15. Houlind MB, Andersen AL, Treldal C, et al. A collaborative medication review including deprescribing for older patients in an emergency department: A longitudinal feasibility study. J Clin Med. 2020;9(2):348. https://doi.org/10.3390/jcm9020348.
16. Farhat A, Al-Hajje A, Lang PO, et al. Impact of Pharmaceutical Interventions with STOPP/START and PIM-Check in Older Hospitalized Patients: A Randomized Controlled Trial. Drugs Aging. 2022;39(11):899-910. https://doi.org/10.1007/s40266-022-00974-7.
17. Jovevski JJ, Smith CR, Roberts JL, et al. Implementation of a compulsory clinical pharmacist-led medication deprescribing intervention in high-risk seniors in the emergency department. Acad Emerg Med. 2023;30(4):410-9. https://doi.org/10.1111/acem.14699.
18. Atey TM, Peterson GM, Salahudeen MS, et al. The impact of partnered pharmacist medication charting in the emergency department on the use of potentially inappropriate medications in older people. Front Pharmacol. 2023;14:1273655. https://doi.org/10.3389/fphar.2023.1273655.
19. Reilly C, Buikstra E, Strivens E, et al. Multidisciplinary care of older adults in the emergency department to influence deprescribing in older adults: a cohort study. J Pharm Pract Res. 2024;54(2):125-34. https://doi.org/10.1002/jppr.1896.
20. Elder GA, King K, Willner M, et al. Implementation of geriatric clinical decision support in the emergency department for potentially inappropriate medications. Acad Emerg Med. 2026;33(1):e70059. Epub 2025 May 13. https://doi.org/10.1111/acem.70059.
21. Hellinger BJ, Gries A, Bertsche T, et al. Impact of an Interprofessional Collaboration Between Physicians and Pharmacists on Fall-Risk-Increasing Drugs in Older Patients with Trauma in the Emergency Department. Geriatrics (Basel). 2025;10(2):46. https://doi.org/10.3390/geriatrics10020046.
22. Tricco AC, Lillie E, Zarin W, et al. PRISMA Extension for Scoping Reviews (PRISMA-ScR): Checklist and Explanation. Ann Intern Med. 2018;169(7):467-73. https://doi.org/10.7326/M18-0850.
